# Effect of Cooling Rate on Mechanical Properties, Translucency, Opalescence, and Light Transmission Properties of Monolithic 4Y-TZP during Glazing

**DOI:** 10.3390/ma15124357

**Published:** 2022-06-20

**Authors:** Ji-In Jeong, Hye-Jeong Shin, Yong Hoon Kwon, Hyo-Joung Seol

**Affiliations:** Department of Dental Materials, School of Dentistry, Pusan National University, Yangsan-si 50612, Korea; stop1989@hanmail.net (J.-I.J.); votaress@nate.com (H.-J.S.); y0k0916@pusan.ac.kr (Y.H.K.)

**Keywords:** 4Y-TZP, cooling rate, glazing, mechanical properties, translucency, opalescence

## Abstract

A standard cooling rate has not been established for glazing; therefore, the effects of the cooling rate on the properties of zirconia need to be evaluated to predict outcomes in clinical practice. 4Y-TZP glazed at three different cooling rates was analyzed to estimate the effect of cooling rate during glazing on the mechanical and optical properties. Hardness tests, field-emission scanning electron microscopy analysis, X-ray diffraction analysis, flexural strength measurement, and optical property evaluations were performed. When 4Y-TZP was glazed at a higher cooling rate (Cooling-1) than the normal cooling rate (Cooling-2), there was no significant difference in grain size, flexural strength, average transmittance, and translucency parameters. The hardness was slightly reduced. The opalescence parameter was reduced for the 2.03 mm thick specimens. When 4Y-TZP was glazed at a lower cooling rate (Cooling-3) than the normal cooling rate, there was no significant difference in hardness, grain size, flexural strength, and translucency parameters. In addition, the average transmittance and opalescence parameters were slightly reduced for the 0.52 and 2.03 mm specimens, respectively. The effects of the cooling rate during glazing on the mechanical and optical properties of 4Y-TZP appear to be minimal and clinically insignificant. Therefore, even if the cooling rate cannot be strictly controlled during glazing, the clinical outcomes will not be significantly affected.

## 1. Introduction

In recent years, zirconia has been introduced and widely used for manufacturing dental prostheses with the help of the computer-aided design/manufacturing technology [1,2]. Zirconia prostheses can be broadly classified as bilayer forms, in which porcelain is veneered on the surface of a core made up of zirconia and single-layer forms; the entire prosthesis is composed of monolithic-form zirconia. Among these, the traditional zirconia core + porcelain veneer double-layer structure is aesthetically pleasing. However, there is a risk that the veneering feldspathic porcelain may be damaged because of delamination, chipping, or residual thermal stresses [3,4,5,6]. Zirconia-based ceramics are known to have superior mechanical properties, resulting from the transformation toughening mechanism [2]. Among the various dental zirconia that are widely used, yttria-stabilized tetragonal zirconia polycrystal (Y-TZP) has the highest robustness, and various studies on the mechanical and optical properties of Y-TZP have been reported [7,8,9,10,11,12,13]. Commercially, 4 mol% yttria-stabilized TZP (4Y-TZP) was developed to supplement translucency as compared to 3Y-TZP and complement the unsatisfactory mechanical properties of 5Y-TZP [14].

Translucency refers to the amount of incident light that is transmitted and scattered to an object. Colors with high translucency appear brighter, and translucency decreases with increased scattering [15]. In general, translucency increases as the yttria content increases in yttria-stabilized zirconia [16]. The translucency of zirconia has been reported to increase with increasing sintering temperature [9]. A translucent material acquires a transmitted color by the spectrum it transmits [15]. In the case of multilayered 4Y-TZP, the spectral transmittance increases from the body layer to the enamel layer [10].

Opalescence is the optical phenomena displayed by the mineral opal. Opalescence makes the tooth appear bluish in the reflected colors and orange/brown in the transmitted colors [17]. Human tooth enamel is opalescent, and because of this, shorter wavelengths of visible light are scattered [18]. Improving the opalescence of dental zirconia leads to esthetic restorations that respond to light similarly to natural human teeth [19]. Kim measured the opalescence parameter of 3 and 5 mol% yttria-stabilized zirconia (A2 shade) after sintering at 1500 °C for 2 h [11]. In the report, the opalescence parameter decreased with increasing yttria content.

The function of a spectrophotometer is to measure the amount of light reflected at each wavelength [15]. The spectral transmittance and reflectance can be recorded with a spectrophotometer. Various parameters (color, translucency, opalescence) for the materials can be calculated from the spectral response. In the dental field, spectrophotometric measurements have been used to evaluate the optical properties for restorative resins, denture teeth, various dental ceramics such as glass ceramic, feldspathic porcelain and zirconia, etc. [9,10,15,16].

When manufacturing various types of ceramic dental prostheses, the glazing treatment is considered as a representative method for providing and maintaining aesthetics and cleanliness to the dental prostheses. That is, by providing gloss and lubricity to the surface of the prosthesis, the prosthesis can harmonize with the existing teeth and surrounding tissues and improve aesthetics. Further, glazing is advantageous for maintaining a clean oral environment by preventing plaque deposition. A thin glass film is formed by glazing after ceramic firing. Using this method, it is possible to supplement the brittleness, which is the biggest weakness in dental ceramic prostheses [20]. The glazed zirconia materials have been reported to have higher values of flexural strength and fracture toughness than the grinded and polished zirconia without glazing treatment [21]. Manawi et al., compared the effects of grinding, finishing, and glazing performed on zirconia core material on the flexural strength and fracture toughness [21]. The glazed samples had higher values of bending strength and fracture toughness than the grinded and polished samples without glazing treatment [21]. However, several studies have reported that it is difficult to increase the flexural strength of dental prostheses via glazing itself; a decrease in the flexural strength was noted after glazing compared to that before glazing [20,22,23]. According to the findings reported by Kumchai et al., this decrease was caused by the pastes used for glazing the zirconia specimens [20]. They reported that glazing using glaze pastes reduced the flexural strength of the zirconia specimens. In contrast, the flexural strength of zirconia specimens was not affected when glazing was simulated without using glaze pastes [20].

The cooling rate during glazing is provided by the manufacturer of the glazing product. Normal glazing schedules specify a cooling time of 3–4 min from the highest temperature to the start temperature, whereas some glazing schedules do not specify the cooling rate [24]. As such, no standard cooling rate has been established for glazing; therefore, the effects of the cooling rate on the various properties of zirconia must be evaluated to predict outcomes in clinical practice. Although the effects of glazing on the mechanical properties of zirconia have been widely reported [20,21,22,23], the effects of varying the cooling rate during glazing on the mechanical and optical properties of 4Y-TZP have not yet been reported. Therefore, this study aimed to analyze the effect of cooling rate during glazing on the mechanical and optical properties of 4Y-TZP. To exclude the effect of glazing pastes, which was experimentally revealed by Kumchai et al. [20], glazing was performed without applying glazing pastes. The null hypothesis is that there is no difference in the mechanical and optical properties corresponding to the changes in the cooling rate during glazing.

## 2. Materials and Methods

### 2.1. Preparation of Specimens

A pre-sintered zirconia block (Ceramill Zolid HT + Preshades: A2 shade, Amann Girrbach, Koblach, Austria) containing Y_2_O_3_ (6.0–7.0 wt%), HfO_2_ (≤5 wt%), Al_2_O_3_ (≤0.5 wt%), and other oxides (≤1 wt%) was cut using a high-speed ultra-precision cutter equipped with a diamond wheel (Accutom-100, Struers, Cleveland, OH, USA). The cut specimens were sintered for 2 h at 1450 °C in a sintering furnace (Ceramill Therm, Amann Girrbach, Koblach, Austria) according to the manufacturer’s instructions. The final sizes of specimens were 10.0 mm × 10.0 mm × 1.0 mm for the hardness test (n = 5/group), X-ray diffraction (XRD) analysis (n = 1/group), and field-emission scanning electron microscopy (FE-SEM) measurement (n = 2/group). The specimen for the flexural strength measurement had a final size of 16.0 mm × 4.06 mm (±0.03) × 1.63 mm (±0.03) (n = 30/group, ISO 6872). The specimens for measuring the optical properties had a final size of 10.0 mm × 10.0 mm with a thicknesses = 0.52 (±0.005), 1.01 (±0.012), 1.52 (±0.005), and 2.03 mm (±0.010) (n = 20/group).

### 2.2. Glazing Heat Treatment

The sintered specimen was glazed without using a glazing paste in a porcelain furnace (Multimat 2 Touch, Dentsply, Bensheim, Germany) to analyze the effect of the cooling rate on zirconia itself without the effect of glaze pastes. The glazing schedule (Table 1) was as provided by the manufacturer of the glazing paste (CZR-E Glaze, Kuraray Noritake Dental Inc., Nagoya, Japan) for zirconia, and it corresponded to the normal glazing schedule with a specific cooling time (4 min). For glazing, the specimens were heated at 850 °C and cooled at three different cooling rates (Table 2) to a starting temperature (600 °C) and then bench-cooled to room temperature. The specimens were divided into three groups according to the cooling rate. In the Cooling-1 group, after heating to 850 °C, the specimens were immediately removed from the porcelain furnace (no cooling time); in the Cooling-2 group, the specimens were cooled to 600 °C by moving the firing chamber gradually to the upper end position (4 min, manufacturer’s suggestion); and in the Cooling-3 group, the specimens were cooled to 600 °C by keeping the firing chamber closed (7 min). In addition, the group that did not proceed with the glazing process was marked as “Before Glazing”.

### 2.3. Hardness Test

The specimens were polished using a 2000-grit silicon carbide abrasive paper (Starcke GmbH & Co. KG, Melle, Lower Saxony, Germany) and then mirror-polished using a 1-µm alumina paste (R&B Inc., Daejeon, Daejeon, Korea). The Vickers hardness of the specimens was measured using a microhardness tester (MVK-H1, Akashi Co., Kawasaki, Japan) under a load of 1 kgf and a load time of 10 s. Five specimens in each group were measured seven times, and the average value and standard deviation were calculated.

### 2.4. FE-SEM Observation

The specimens were sputter-coated with platinum for 120 s. The microstructures of the specimens were observed at an acceleration voltage of 10 kV using FE-SEM (JSM-7200F, Jeol, Akishima, Japan). Using Image J Software (version 1.53e, National Institute of Health, Bethesda, USA), 600 grains were used to measure the sizes in six FE-SEM images for each group, and the average value and standard deviation were calculated.

### 2.5. XRD Analysis

The crystal structures of the specimens were analyzed at a voltage of 40 kV, tube current of 30 mA, and step size of 0.013° with Ni-filtered CuKα radiation using high-resolution XRD (X’Pert^3^ Powder; PANalytical, Amsterdam, The Netherlands).

### 2.6. Flexural Strength Measurement

A three-point flexural strength test was conducted using a universal testing machine (Instron 3345, Norwood, MA, USA) according to the ISO 6872:2019 standard. The distance between the supports was 12.0 mm, and the crosshead speed was 0.5 mm/min. The flexural strength was calculated using the following formula [25]:

*σ* = 3*Nl*/2*bd*^2^ (1)

where *σ*: flexural strength, *N*: fracture load (in N), *l*: distance between the supports (in mm), *b*: width of the specimen (in mm), and *d*: thickness of the specimen (in mm). The Weibull modulus (*m*) and characteristic strength (*σ*_0_) were obtained by the median ranking and maximum likelihood method using reliability analysis software (Reliability and Maintenance Analyst v5.0.9, Engineered Software, Inc., Lacey, WA, USA). The Weibull distribution was determined using the following formula [25]:

(2) $$\mathrm{Pf}=1-\exp[{-(\frac{\sigma }{\sigma_{0}})}^{m}]$$

where Pf = fracture probability = (rank − 0.3)/(N + 0.4), N=number of samples, *σ* = flexural strength, *σ*_0_ = characteristic strength (the strength corresponding to a probability of failure of 63.2%), and *m* = Weibull modulus.

### 2.7. Optical Properties Evaluation

The specimens were dry-polished up to 5000-grit SiC abrasive paper before sintering. The spectral transmittance and reflectance were recorded with a spectrophotometer (CM-3600d, Konica Minolta Sensing, Osaka, Japan) according to CIE standard light source D65 and 2° standard observer. The measurement was conducted three times at intervals of 10 nm from 360 to 740 nm (n = 5/group). The light transmittance was obtained as a percentage value between 100% (transparent) and 0% (opaque) by dividing the overall light transmittance by the overall light transmittance without the specimen in the spectrophotometer. The spectral reflectance was measured in the reflectance mode after placing specimens on white and black backgrounds in a mode including ultraviolet light. Average transmittance (AT) was calculated by dividing the sum of transmittance (%) at each wavelength with the number of data points. The translucency parameter (TP) was calculated using the following formula:

TP = [(L*_W_ − L*_B_)^2^ + (a*_W_ − a*_B_)^2^ + (b*_W_ − b*_B_)^2^]^1/2^ (3)

where W and B are the color coordinates on white and black background, respectively [26,27].

The opalescence parameter (OP) was calculated using the following formula:

OP = [(a*_T_ – a*_R_)^2^ + (b*_T_ – b*_R_)^2^]^1/2^ (4)

where T is the transmitted light and R is the reflected light on a black background [26,27].

The relationship between the thickness of each specimen and the AT and TP values was analyzed using the regression analysis of an exponential function [28]:

*y* = *a* × exp^(*bx*)^ (5)

where *y*: observed AT or TP value, *x*: specimen thickness, and *a*, *b*: constant.

### 2.8. Statistical Analysis

The results were analyzed using the statistical program (Statistical Product and Service Solutions 25.0, IBM Co., Armonk, NY, USA), and the statistical significance level was 0.05. The normality was analyzed by the Shapiro–Wilk test. The results were analyzed by one-way ANOVA and post hoc Tukey’s HSD test (hardness and flexural strength) and by two-way ANOVA and post hoc Tukey’s HSD test (AT and TP). The grain size and OP value did not satisfy the normality distribution; therefore, the results were analyzed by the Kruskal–Wallis H test and the generalized linear model, respectively.

## 3. Results

### 3.1. Hardness

In Table 3, the hardness of Cooling-1 was lower than that of the Cooling-2 and -3 groups (*p* = 0.001); however, the difference was negligible.

### 3.2. FE-SEM Analysis

**Figure 1:**
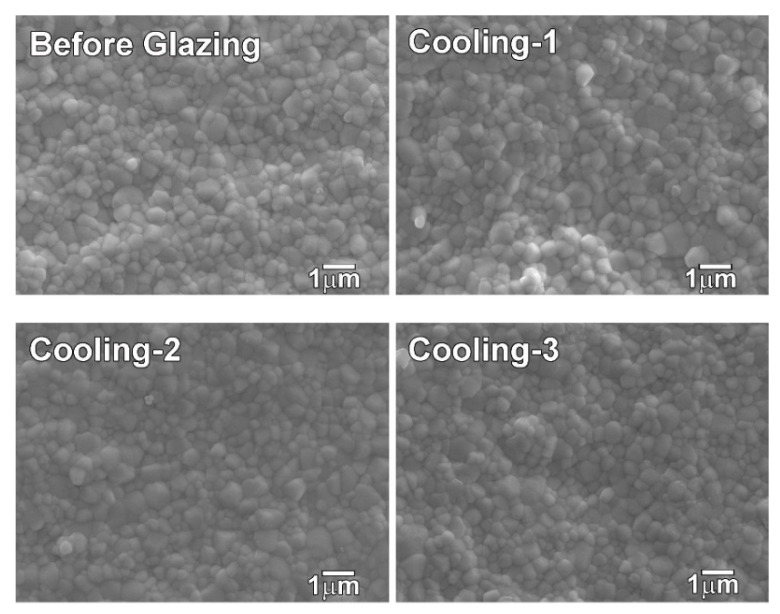
Microstructures of each group of zirconia specimens (×10,000).

The FE-SEM analysis results (Figure 1) indicated that equiaxed crystal structures were present in all the groups, and all of them had similar microstructures. The average grain size (Table 3) of the groups cooled at various rates during glazing (Cooling-1, Cooling-2, and Cooling-3) did not show a significant difference (*p* > 0.05). In addition, the average grain size of the Before Glazing group was not different from that of the Cooling-1, Cooling-2, and Cooling-3 groups (*p* > 0.05).

### 3.3. XRD Analysis

**Figure 2:**
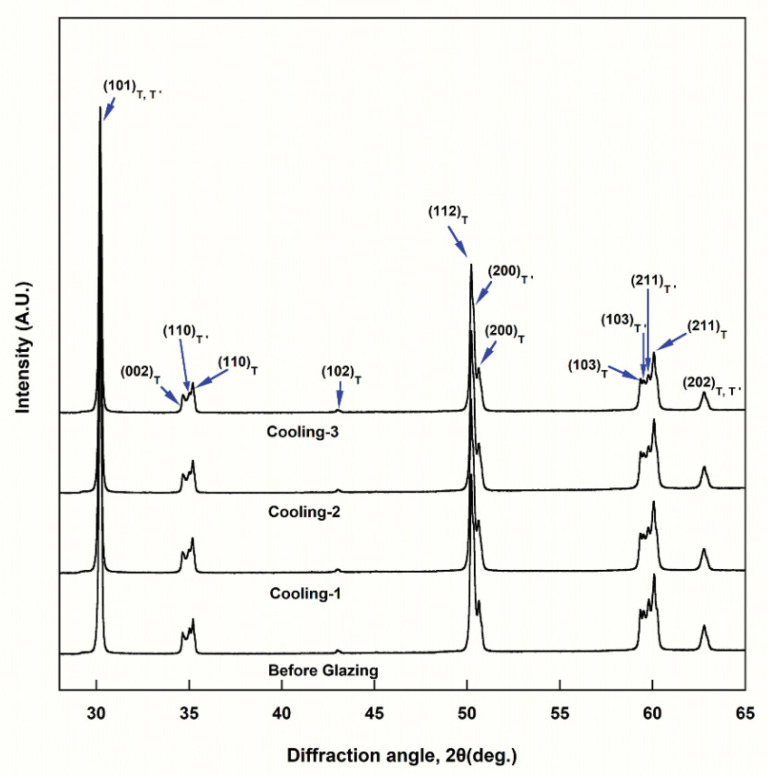
XRD patterns for each group of zirconia specimens.

The XRD analysis (Figure 2) showed that a tetragonal phase (T) and metastable phase (T′) of the tetragonal phase coexisted in all the groups; the cubic or monoclinic phase was not observed. The lattice constants of T′ were a = 3.623 Å and c = 5.159 Å, and those of T were a = 3.602 Å and c = 5.172 Å.

### 3.4. Flexural Strength

The flexural strength (Table 3) did not show a significant difference with respect to the cooling rate during glazing. In the Weibull analysis, the highest value of the characteristic strength (913.72 MPa) was obtained for the Cooling-1 group, while the lowest value (857.59 MPa) was obtained for the Cooling-2 group. Table 3 shows a Weibull modulus of 7.17 (Cooling-1), 7.37 (Cooling-2), and 8.14 (Cooling-3). These values are close, with overlapping confidence intervals.

### 3.5. Spectral Transmittance and Average Transmittance (AT)

**Figure 3:**
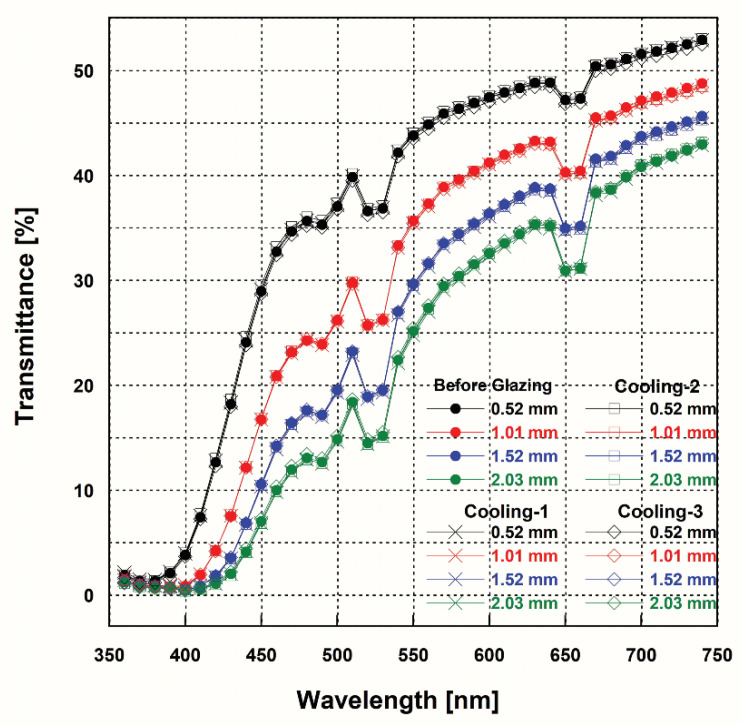
Spectral transmittance curves for each group of zirconia specimens.

The spectral transmittance (Figure 3) showed similar results in all the groups. The average transmittance (AT) values were obtained from the spectral transmittance and analyzed statistically (Table 4). A difference in the AT value corresponding to the cooling rate was observed only at the thickness of 0.52 mm; when 4Y-TZP was glazed at a lower cooling rate (Cooling-3) than the normal cooling rate (Cooling-2), the average transmittance reduced by 1.5% at the thickness of 0.52 mm (*p* = 0.004). As the thickness of the specimen increased from 0.52 to 2.03 mm, the AT value decreased from approximately 36 to 22 in all the groups (*p* < 0.001). In each group, there was no interaction between the thickness and the cooling rate during glazing (*p* > 0.05). Table 5 lists the results of the regression analysis of the relationship between the AT and thickness for each group of the zirconia specimens using Equation (5). In the regression equation between each group, the values of *a* and *b* were similar (Table 5).

### 3.6. Translucency Parameter (TP)

The TP values were obtained from the spectral reflectance and analyzed statistically (Table 6). There was no significant difference in the TP value in all the groups (*p* > 0.05). The decrease in the TP value was significant as the thickness increased (*p* < 0.001). There was no interaction between the thickness and cooling rate during glazing in each group (*p* > 0.05). Table 7 lists the results of the regression analysis between the TP and thicknesses using Equation (5). In the regression equation between each group, the values of *a* and *b* were similar (Table 7).

### 3.7. Opalescence Parameter (OP)

The OP values of the specimens with different thicknesses at different cooling rates during glazing were obtained and analyzed statistically (Table 8). The OP values of the Cooling-1 and Cooling-3 groups were lower by 0.99 (*p* = 0.011) and 0.93 (*p* = 0.017) than that of the Cooling-2 group, respectively, at the thickness of 2.03 mm. As the thickness increased, the OP value increased in all the groups (*p* < 0.001). There was no interaction between the thickness and cooling rate during glazing in each group (*p* > 0.05).

## 4. Discussion

In this study, the mechanical and optical properties and crystal structure of 4Y-TZP (A2 shade) glazed at three different cooling rates were analyzed. The null hypothesis, which predicted that there would be no difference in the mechanical and optical properties of 4Y-TZP corresponding to the cooling rate during glazing was partially accepted. Based on the hardness test, the hardness value for Cooling-1 was lower than those for the other groups (*p* = 0.001); however, the difference was minimal (Table 3). The grain size analysis results revealed that no significant difference was observed with the cooling rate (Table 3). In a study conducted with 3Y-TZP, the grain size after sintering for 2 h at 1400–1500 °C was smaller than the value obtained in this study [29]. In general, with an increase in the yttria content, the grain size of yttria-stabilized zirconia increases [30]. In addition, it has been reported that the grain size of zirconia shows a positive correlation with the final sintering temperature [31].

The XRD analyses indicated that the cooling rate during glazing did not affect the crystal structure of the specimens. In all the groups, only the tetragonal phase (T) was observed. In particular, the metastable tetragonal phase (T′) coexisted with the tetragonal phase. The lattice constants of T′ were a = 3.623 Å and c = 5.159 Å, while those of T were a = 3.602 Å and c = 5.172 Å. Therefore, the lattice parameter (a) of T′ was marginally greater than that of T, and the lattice parameter (c) was marginally lower than that of T. Thus, the axial ratio (*c/a* ratio = c/√2a) of T′ (1.0069) was closer to one than that of T (1.0153). These values are very similar to those reported by Kim [16]. It has been reported that the presence of the metastable tetragonal phase (T′) instead of the tetragonal phase (T) improves the translucency of the zirconia, as the light scattering due to birefringence decreases because its lattice parameters are closer to that of the cubic phase [16]. Kim reported that when zirconia containing 3–5 mol% yttria is sintered at 1550 °C and is then rapidly cooled, the generation of T′ increases, thus increasing the translucency of the material as compared to the conventional cooling process [16].

The flexural strength did not show a significant difference with respect to the cooling rate during glazing (Table 3). The Weibull characteristic strength corresponds to the strength when the failure probability is 63.2% [32]. In the Weibull analysis, the highest value of the characteristic strength (913.72 MPa) was obtained for the Cooling-1 group, while the lowest value (857.59 MPa) was obtained for the Cooling-2 group. However, these values are close, with overlapping confidence intervals. The differences in the Weibull modulus for the flexural strength were not significant. Therefore, it is considered that the reliability of the flexural strength does not change, regardless of the change in the cooling rate in 4Y-TZP. In the case of 3Y-TZP, as the cooling rate during glazing increased, the Weibull modulus for the flexural strength tended to decrease [33]. In several studies that have focused on the change in flexural strength during the glazing of zirconia, it has been reported that the flexural strength decreases after the glazing process [20,22,23]. This decrease was caused by the glaze pastes [20]. Kumchai et al., reported that the flexural strength of zirconia specimens was not affected when glazing was simulated without using glaze pastes [20]. In this study, conducted without applying a glazing paste, the flexural strength did not change significantly after glazing in all the groups with different cooling rates. In the case of 3Y-TZP, which has lower yttria content, the flexural strength value was reported to be >900 MPa [34]. This was higher than the flexural strength of the 4Y-TZP tested in this study. To analyze the translucency of the specimens used in this study, the spectral transmittance and the translucency parameter (TP) were measured. All the groups showed similar spectral transmittance curves. The spectral transmittance changed according to the wavelength and thickness of the specimen in all the groups [10,35,36]. The spectral transmittance decreased at ~520 and 650 nm and then increased again. This trend has been reported for other pre-colored zirconia products [16] but not for uncolored zirconia products [33,35]. This decrease in the spectral transmittance of pre-colored zirconia in a specific wavelength range may be attributed to the presence of the pigment [10].

The average transmittance (AT) value in the Cooling-2 group was higher than that in the Cooling-3 group at the thickness of 0.52 mm (*p* = 0.004), although the difference was negligible (Table 4). The AT decreased significantly as the thickness of the specimen increased in all the groups (*p* < 0.001). The regression analysis showed that the AT changed exponentially with the thickness. The spectral transmittance of a uniform material is given as an exponential function of the thickness of the material (T = exp^(−αx)^) [37]. In this equation, α represents the linear attenuation coefficient. Attenuation occurs because of the interaction between the light and material via scattering or absorption [37]. As the linear attenuation coefficient (α) increases, the degree of scattering or absorption of light increases, and the opacity of the object increases [37]. Shiraishi and Watanabe reported that the AT of a nonuniform material, Ce-TZP/alumina nanocomposite, varies exponentially with its thickness [35], although the materials were not homogeneous in microstructure. In their report, the absolute value of *b* in Equation (5) that corresponded to the linear attenuation coefficient (α) was 4.267, which is apparently higher than the value in Table 5 (0.321–0.338) owing to its opacity [35]. Cekic-Nagas measured the mean light transmittance (%) for 3Y-TZP of an A1 shade with different thicknesses (0.3, 0.5, and 0.8 mm) [36]. In the report, the spectral transmittance decreased according to the thickness of the specimen. The reported AT value (34–38% at 0.5 mm) is similar to the value obtained in this study (~36% at 0.52 mm), even with lower yttria content than that in this study [36]; this is because the 3Y-TZP used has a lighter shade. In addition, in the case of uncolored 3Y-TZP, the reported AT values (29.80% and 28.61%) at 1.01 mm were similar to the values obtained in this study (~29% at 1.01 mm), even with lower yttria content [13]. This can be attributed to the fact that the uncolored 3Y-TZP contains no pigment, whereas the 4Y-TZP used in this study was of the A2 shade. In the case of multilayered 4Y-TZP with a shade gradation from A1.5 to A2, the spectral transmittance increased from the body layer to the enamel layer [10]. In the report, the AT value for the enamel layer (~32% at 1.0 mm) was slightly higher than the value obtained in this study (~29% at 1.01 mm), owing to its lighter shade.

Translucency refers to the amount of incident light that is transmitted and scattered to an object. In this study, there was no significant difference in the translucency parameter (TP) according to the cooling rate (*p* > 0.05, Table 6). As the thickness increased, the TP value decreased exponentially (*p* < 0.001). In general, translucency increases as the yttria content increases in yttria-stabilized zirconia [16]. In the case of uncolored 3Y-TZP, the TP value was 15.88 at 1.0 mm [38], which was higher than the values presented in Table 6 (~12.4 at 1.01 mm), owing to the absence of pigment. In the case of multilayered 5Y-TZP (upper lighter layer of Shade 0/B1), the TP value was 19.41 at 1.0 mm [38], which was higher than the values in Table 6 (~12.4 at 1.01 mm), owing to the lighter shade and the higher yttria content.

Human tooth enamel is opalescent, and because of this, shorter wavelengths of visible light are scattered [18]. The opalescence parameter (OP) for human enamel is known to be 19.8–27.6 at the thickness of 0.9–1.3 mm [19]. In this study (Table 8), OP was ~19 at the thickness of 1.01 mm, and OP increased parabolically from approximately 11 to 30 with increasing thickness (0.52 mm to 2.03 mm), regardless of the cooling rate. From the above, it was determined that as the thickness increased, the OP increased, while the AT and TP values decreased. Kim measured the OP value of 3Y-TZP and 5Y-PSZ (at 1 mm, A2 shade) after sintering at 1500 °C for 2 h [11]. In the report, as the yttria content increases, the OP decreases; the OP value was ~17.5 for 3Y-TZP and ~13 for 5Y-PSZ.

The cooling rate during glazing is provided by the manufacturer of the glazing product. However, some glazing schedules do not specify the cooling rate [24]. As such, no standard cooling rate has been established for glazing; therefore, the effects of the cooling rate during glazing on the mechanical and optical properties of 4Y-TZP must be evaluated to predict outcomes in clinical practice. On the basis of the results discussed thus far, the effects of the cooling rate during glazing on the mechanical and optical properties of 4Y-TZP appear to be minimal and clinically insignificant. Therefore, even if the cooling rate cannot be strictly controlled during glazing, the clinical outcomes will not be significantly affected. The novelty of this study is investigation of the effects of varying the cooling rate during glazing on the mechanical and optical properties of 4Y-TZP; this has not yet been reported. In this study, glazing treatment was performed without applying glazing pastes, to exclude the effect of glazing pastes [20]. The limitation of this study is that the experiment was conducted only with one kind of 4Y-TZP, without applying a glazing paste. Therefore, the results may not be identical for other commercial 4Y-TZP products applied with glazing pastes. Further studies on the effect of cooling rate during glazing on the mechanical and optical properties of various 4Y-TZP products applied with glazing paste are needed to further confirm our findings.

## 5. Conclusions

When 4Y-TZP was sintered and glazed at a cooling rate higher or lower than the normal cooling rate suggested by the manufacturer, the difference in flexural strength, grain size, and translucency parameters with respect to the cooling rate during glazing was not statistically significant. The average transmittance for Cooling-3 was slightly lower compared to that for Cooling-2 (for 0.52 mm). The opalescence parameters for Cooling-2 was slightly higher than those for the other groups (for 2.03 mm).

## Figures and Tables

**Table 1:** Glazing schedule.

Pre- Drying (min)	Heat Rate (°C/min)	Start Temp. (°C)	Final Temp. (°C)	Hold Time (min)	Vacuum Level (cm/HG)	Start Vacuum (°C)	Vacuum Release(°C)
5	65	600	850	1	72	600	850

**Table 2:** Cooling rate during glazing.

Cooling Rate	Cooling-1	Cooling-2	Cooling-3
°C/min	>250	62.5	35.7
Condition	Firing chamber moves immediately to upper end position	Firing chamber moves gradually to upper end position	Firing chamber remains closed
Cooling time	0 min (no cooling time)	4 min (manufacturer’s suggestion)	7 min (extended cooling time)

**Table 3:** Hardness value, grain size, flexural strength, characteristic strength, and Weibull modulus for each group of the zirconia specimens.

Code	Mean Hardness±SD (HV)	Mean Grain Size ±SD (μm)	Mean Flexural Strength ±SD (MPa)	Characteristic Strength, *σ*_0_ (MPa) (95% Confidence Interval)	Weibull Modulus, *m* (95% Confidence Interval)
Before Glazing	1401.06^b^ (4.89)	0.474^a^ (0.18)	849.58^a^ (118.77)	900.03 (860.14–941.77)	8.34 (6.31–11.01)
Cooling-1	1396.77^a^ (5.79)	0.479^a^ (0.15)	855.33^a^ (139.30)	913.72 (866.77–963.21)	7.17 (5.41–9.50)
Cooling-2	1400.23^b^ (4.07)	0.474^a^ (0.15)	805.47^a^ (121.73)	857.59 (814.60–902.84)	7.37 (5.61–9.69)
Cooling-3	1400.54^b^ (4.43)	0.467^a^ (0.14)	838.41^a^ (119.83)	888.71 (848.41–930.92)	8.14 (6.18–10.72)

SD: standard deviation. The same lowercase letter in the same column indicates no statistically significant difference among groups (*p* > 0.05).

**Table 4:** Average transmittance (AT, %) for each group of the zirconia specimens with different thicknesses (mean ± SD).

Code	Before Glazing	Cooling-1	Cooling-2	Cooling-3
0.52 mm	36.10^Dab^ (0.24)	36.14^Dab^ (0.20)	36.32^Db^ (0.22)	35.76^Da^ (0.22)
1.01 mm	29.28^Ca^ (0.24)	29.16^Ca^ (0.48)	29.20^Ca^ (0.24)	29.11^Ca^ (0.25)
1.52 mm	24.96^Ba^ (0.13)	24.78^Ba^ (0.24)	24.81^Ba^ (0.19)	24.90^Ba^ (0.17)
2.03 mm	21.88^Aa^ (0.20)	21.74^Aa^ (0.62)	21.83^Aa^ (0.40)	22.06^Aa^ (0.28)

The same uppercase letter indicates no statistically significant difference among thicknesses (*p* > 0.05), and the same lowercase letter indicates no statistically significant difference among groups (*p* > 0.05).

**Table 5:** Regression analysis of the relationship between the average transmittance (AT) and thickness for each group of the zirconia specimens.

Code	Regression Equation *y* = *a* × exp^(*bx*)^ (5)	R^2^	*p*
Before Glazing	*y* = 41.77exp^(−0.332*x*)^	0.988	<0.001
Cooling-1	*y* = 41.87exp^(−0.338*x*)^	0.981	<0.001
Cooling-2	*y* = 42.01exp^(−0.338*x*)^	0.983	<0.001
Cooling-3	*y* = 41.08exp^(−0.321*x*)^	0.984	<0.001

R^2^: correlation coefficient.

**Table 6:** Translucency parameter (TP) for each group of the zirconia specimens with different thicknesses (mean ± SD).

Code	Before Glazing	Cooling-1	Cooling-2	Cooling-3
0.52 mm	17.43^Da^ (0.27)	17.21^Da^ (0.19)	17.40^Da^ (0.22)	17.28^Da^ (0.21)
1.01 mm	12.54^Ca^ (0.12)	12.51^Ca^ (0.34)	12.34^Ca^ (0.15)	12.42^Ca^ (0.12)
1.52 mm	8.32^Ba^ (0.12)	8.44^Ba^ (0.15)	8.52^Ba^ (0.14)	8.35^Ba^ (0.16)
2.03 mm	5.40^Aa^ (0.20)	5.65^Aa^ (0.35)	5.34^Aa^ (0.16)	5.29^Aa^ (0.40)

The same uppercase letter indicates no statistically significant difference among thicknesses (*p* > 0.05), and the same lowercase letter indicates no statistically significant difference among groups (*p* > 0.05).

**Table 7:** Regression analysis of the relationship between the translucency parameter (TP) and thickness for each group of the zirconia specimens.

Code	Regression Equation *y* = *a* × exp^(*bx*)^ (5)	R^2^	*p*
Before Glazing	*y* = 26.57exp^(−0.786*x*)^	0.994	<0.001
Cooling-1	*y* = 25.62exp^(−0.748*x*)^	0.991	<0.001
Cooling-2	*y* = 26.44exp^(−0.783*x*)^	0.993	<0.001
Cooling-3	*y* = 26.53exp^(−0.792*x*)^	0.988	<0.001

R^2^: correlation coefficient.

**Table 8:** Opalescence parameter (OP) for each group of the zirconia specimens with different thicknesses (mean ± SD).

Code	Before Glazing	Cooling-1	Cooling-2	Cooling-3
0.52 mm	11.14^Aa^ (0.22)	10.74^Aa^ (0.30)	10.90^Aa^ (0.42)	11.32^Aa^ (0.41)
1.01 mm	19.35^Ba^ (0.36)	19.07^Ba^ (0.63)	19.36^Ba^ (0.67)	19.38^Ba^ (0.39)
1.52 mm	26.02^Ca^ (0.71)	25.57^Ca^ (0.54)	26.10^Ca^ (0.46)	26.22^Ca^ (0.47)
2.03 mm	30.28^Dab^ (0.52)	29.71^Da^ (1.06)	30.70^Db^ (1.44)	29.77^Da^ (0.74)

The same uppercase letter indicates no statistically significant difference among thicknesses (*p* > 0.05), and the same lowercase letter indicates no statistically significant difference among groups (*p* > 0.05).

## Data Availability

Not applicable.
